# Silicon and Plant Natural Defenses against Insect Pests: Impact on Plant Volatile Organic Compounds and Cascade Effects on Multitrophic Interactions

**DOI:** 10.3390/plants8110444

**Published:** 2019-10-23

**Authors:** Nicolas Leroy, Félix de Tombeur, Yseult Walgraffe, Jean-Thomas Cornélis, François J. Verheggen

**Affiliations:** 1 Gembloux Agro-Bio Tech, TERRA, University of Liège, Avenue de la Faculté d’Agronomie 2, 5030 Gembloux, Belgium; nleroy@uliege.be (N.L.); yseultwalgraffe@hotmail.com (Y.W.); 2 Water-Soil-Plant Exchanges, Gembloux Agro-Bio Tech, University of Liège, Avenue Maréchal Juin 27, 5030 Gembloux, Belgium; felix.detombeur@uliege.be (F.d.T.); jtcornelis@uliege.be (J.-T.C.)

**Keywords:** silicon, attraction, chemical cues, insect-plant interactions, biotic stressors, VOCs

## Abstract

Environmental factors controlling silicon (Si) accumulation in terrestrial plant are key drivers to alleviate plant biotic stresses, including insect herbivory. While there is a general agreement on the ability of Si-enriched plant to better resist insect feeding, recent studies suggest that Si also primes biochemical defense pathways in various plant families. In this review, we first summarize how soil parameters and climate variables influence Si assimilation in plants. Then, we describe recent evidences on the ability of Si to modulate plant volatile emissions, with potential cascade effects on phytophagous insects and higher trophic levels. Even though the mechanisms still need to be elucidated, Si accumulation in plants leads to contrasting effects on the levels of the three major phytohormones, namely jasmonic acid, salicylic acid and ethylene, resulting in modified emissions of plant volatile organic compounds. Herbivore-induced plant volatiles would be particularly impacted by Si concentration in plant tissues, resulting in a cascade effect on the attraction of natural enemies of pests, known to locate their prey or hosts based on plant volatile cues. Since seven of the top 10 most important crops in the world are Si-accumulating Poaceae species, it is important to discuss the potential of Si mobility in soil-plant systems as a novel component of an integrated pest management.

## 1. Introduction

Through its high abundance and relative high mobility in terrestrial environments, silicon (Si) is an ubiquitous element in the soil-plant system [1,2,3]. The role of Si in plant development has been widely debated but is nowadays considered as non-essential (or quasi-essential) for plant growth [4]. Accumulating evidences from the last two decades suggests that Si could mitigate plant biotic stresses, including microbial agents (viruses, bacteria and fungi) and animals (vertebrate and arthropod herbivores) [5,6].

Silicon can physically and biochemically impact plants:

(1) Si accumulates as amorphous hydrated silica (SiO_2_·nH_2_O) in the plant epidermal cells [7,8,9], increasing the hardness and abrasiveness of plant tissues, consequently impacting oral parts of phytophagous insects and reducing food intake [10,11]. Si-enriched tissues also show a reduced digestibility and palatability, leading to a decrease in insect’s growth rate [12,13,14]. Si also impacts the feeding behavior of phytophagous insects, as observed in *Spodoptera exempta* Walker and *Schistocerca gregaria* Forsskål, that avoid grass plants enriched with Si [15]. In Si-accumulator species, foliar Si accumulation is the main factor responsible of plant resistance against herbivory. There is now evidences that Si, in non-accumulator species, induces changes in plant nutritional quality and reduce performance of insect herbivore [16];
(2) Silicon is also involved in the biochemical response of plants against the attack of a phytophagous insect [17]. Plants characterized by high concentration of Si show higher expression of genes encoding for defensive enzymes, including phenylalanine ammonia lyase, lipoxygenase and polyphenol oxidase [18,19]. This results in higher levels of defensive compounds, such as phenolics or phytoalexins [20,21].

**Figure 1:**
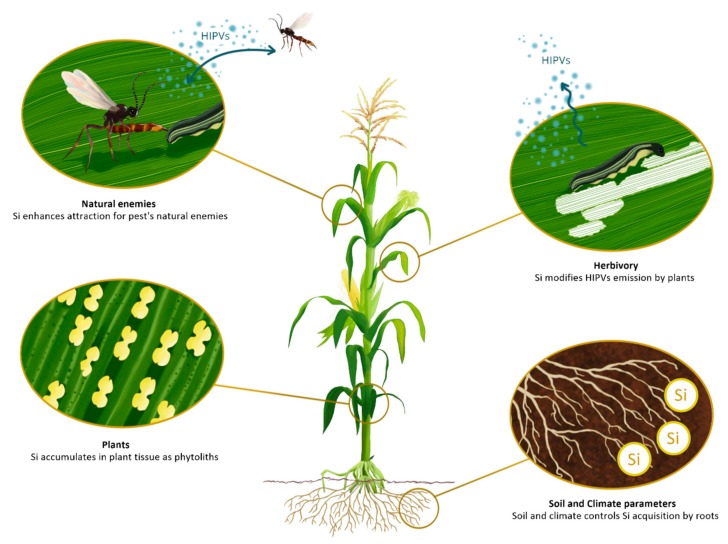
Soil and climatic parameters control silicon (Si) assimilation by terrestrial plants. Si is available in the soil solution as uncharged monomeric monosilicic acid (H_4_SiO_4_) and plants accumulate Si as amorphous hydrated silica (SiO_2_·nH_2_O, called phytoliths) in the epidermal cells. Herbivore-Induced Plant Volatiles (HIPVs) emissions by plants are modified by Si, that in turn affect tritrophic interactions. Drawing by Levicek Carolina & Nguyen Juliette.

In this review, we aim at linking two Silicon-related groups of research studies (Figure 1). First, we summarize how soil parameters and climate variables influence Si assimilation in plants. Secondly, we describe recent evidences on the ability of Si to modulate plant volatile emissions, with potential cascade effects on phytophagous insects and higher trophic levels [22,23]. The mechanisms by which Si affects plant physiology are not clear and are a current matter of debate. Si was suggested to interact with plant defensive pathways as a secondary messenger [24] or to improve plant defenses through biological silicification via the formation of a silica obstruction [25]. The first hypothesis was, however, undermined by the work of Vivancos et al. [26], using *Arabidopsis* mutants that are able to accumulate large amounts of Si and deficient in the activation of the salicylic acid pathway (SA). In the second hypothesis, Si is described as an extracellular prophylactic agent against biotic stresses. The amorphous Si portion that deposits in the apoplast could interfere with the recognition process occurring upon infection [27,28].

We finally discuss the potential of Si fertilization as a novel component of integrated pest management strategies.

We conducted a comprehensive search of the literature using Scopus^®^ (Elsevier), PubMed^®^, and Google Scholar^®^ and combining the following queries: silicon, plants, phytoliths, herbivores, insects, volatile organic compounds (VOCs), herbivore-induced plant volatiles (HIPVs), multitrophic interactions. We also focused on the literature focusing on the impact of climatic and soil parameters on silicon accumulation in plants using the following keywords: plant available silicon, weathering, pH, climate change, soils, carbon dioxide, temperature, monosilicic acid. This research was conducted between December 2018 and September 2019 and the resulting references were selected or rejected based on the abstracts of the published papers.

## 2. Soil and Climate Control Si Accumulation in Plants

Although phylogenetic variation explains most of the highly contrasted Si concentrations in terrestrial plants [29], the environmental factors are key for understanding intra-specific variation [30,31]. In soils, Si is available for micro-organisms and plants as uncharged monomeric monosilicic acid, H_4_SiO_4_, at common soil pH values [32]. Its concentration in soil solution generally ranges from 0.1 to 0.6 mM [33]. Nevertheless, variations of two orders of magnitude have been reported in literature (from 1 to more than 100 mg of Si per kg of soil in CaCl_2_-extractable solution [34,35]). Such differences are mainly governed by soil mineralogy [36]. Indeed, the concentration of plant-available Si decreases with increasing soil weathering degree and decreasing pH, given soil desilicification [32,37,38,39]. Nevertheless, it has been recently highlighted that alkaline pH lead to a decrease of plant-available Si given increasing adsorption of Si on soil mineral surfaces [40]. Soil pH is thence a key driver of Si plant-availability [35]. Besides, the magnitude of Si biocycling in soil-plant systems and the resulting amount of phytoliths returned on topsoil also play a key role in the replenishment of Si in soil solution [36,41]. An integrated understanding of Si mobility in soil-plant systems is therefore essential for optimizing Si benefits in terms of plant protection.

Climate also plays a key role in Si accumulation by plants, mainly through soil water availability and plant evapotranspiration. Ryalls et al. [42] showed that plant Si concentration increases in roots and shoots under elevated rainfall, and thence soil moisture, in *Microlaena stipoides* Robert Brown. Quigley & Anderson [31] made the same observation in *Themeda triandra* Forsskål. Jenkins et al. [43] showed that the number of silicified cells per phytoliths increased with irrigation. Si uptake is thence largely dependent on soil moisture [44,45,46]. Besides soil water availability, the rate of plant evapotranspiration—driven by net irradiance, air density, air heat capacity, vapor pressure deficit and latent heat of vaporization among others-also controls plant Si uptake and concentrations [7,47]. As a result, Henriet et al. [48] showed that Si concentration in different shoot organs of banana plants was directly related to its transpiration rate. Cornélis et al. [49] attributed the higher Si uptake by Douglas fir leaves compared to Black pine leaves to a higher transpiration rate and Euliss et al. [50] showed that the leaf Si concentration of different grasses and wetland species was directly linked to its transpiration rate. Field studies showed that Si uptake increases under drought stress [51,52] as a defense mechanism [53]. Fulweiler et al. [54] observed that anthropogenic changes (elevated atmospheric CO_2_ concentrations) may increase biological Si pumping in forest. Climatic variable (air temperature, CO_2_ concentrations) and pedoclimatic variable (soil water content) are therefore two key properties that must be integrated for better understanding of Si accumulation in plants.

Understanding the drivers of Si accumulation as a natural plant defense is key because seven of the top 10 most widespread crops in the world are Si-accumulating Poaceae species [55], especially in the face of expected climate changes [56] and the lack of resilience of conventional agriculture [57]. Sugarcane crops are concentrated in India, Brazil, China and the Caribbean Islands under tropical/subtropical climate where highly weathered soils lead to low Si plant-availability [36]. In contrast, wheat and barley crops are concentrated in the United States, Europe, Canada and on drier areas of China and India (compared to sugarcane), at more temperate climate. In this environment, soils are less desilicated, leading to higher Si plant-availability. The climate variables in tropical and temperate agro-ecosystems will also strongly affect Si accumulation in plants through very contrasting air temperature and soil water content. The natural ability of plants to accumulate Si is therefore highly variable at the global scale, and far from being understood.

## 3. Trophic Level One: Si in Plants

Under similar conditions, plants do not have the same abilities to accumulate Si. For the plant to benefit from Si, they must first transport it from the soil to the plant tissue [58]. Before any Si transporters have been identified, a classification of plant species based on their Si content was proposed. Three different plant groups, based on their ability to uptake Si and accumulate it in their tissues, were defined: Si *accumulators*, Si *intermediate* and Si *excluders*. Plants known as Si *accumulators* are characterized by active Si uptake that leads to a decrease of Si concentration in soil solution. Plants with an *intermediate* Si content perform passive Si uptake while *excluder* plant species are rejective to Si [9,59]. Poaceae typically contain high concentration of Si (up to 10% of their dry mass), while Fabaceae or Brassicaceae accumulate Si for less than 1% of their dry mass [29,60].

By discovering Si transporters, Ma et al. [61] paved the way for a better understanding of Si accumulation in plants. Two types of Si transporter were identified in plants: channel-type, also named influx channels (Lsi1) and efflux transporters (Lsi2). Channel-type transporters assist the passive transport of Si across the plasma membrane to plant cells. Efflux transporters are implicated in the transport of Si out of the plant cells to the xylem. Both types of transporters were first identified in rice [61,62,63]. Homologs were then isolated in barley (*Hordeum vulgare* L., HvLsi1, HvLsi6, HvLsi2) [64,65], wheat (*Triticum aestivum* L., TaLsi1) [66], maize (*Zea mays* L., ZmLsi1, ZmLsi6, ZmLsi2) [67] and pumkin (*Cucurbita moschata* Duschesne, CmLsi1, CmLsi2) [68,69]. The significant difference of plant ability to accumulate Si is due to different mechanisms of Si uptake by root among plant species. In higher plants, the transport and distribution system of Si is totally based on the cooperation of influx and efflux transport proteins [70,71,72].

Lsi1 belongs to the subfamily Nodulin 26-like intrinsic proteins (NIPs) of aquaporins proteins (AQPs). AQPs, a channel-type protein, facilitate transport of water and/or small uncharged solutes across cell membranes [73]. All Lsi1s from different plant species belong to the NIP III group, which is characterized by a unique selectivity filter comprising four amino acids Glycine (G), Serine (S), Glycine (G) and Arginine (A). Moreover, in typical aquaporins, the predicted amino acid sequence is well conserved and has six transmembrane domains and two Asparagine (Asn)-Proline (Pro)-Alanine (Ala) NPA motifs. Interestingly, Deshmukh et al. [74] showed that the distance between NPA domains is another important key for Si transporters characterization. For example, poor Si accumulators like tomatoes contained 109 amino acids (AAs) between the NPA domains while high accumulators conserved their 108 AAs between the NPA domains. Coskun et al. [25] recently suggested that molecular criteria should be adopted to classify plants as accumulators or non-accumulators of Si. Plant AQPs belonging to the NIP III group with a GSRA selectivity filter and two NPA domains separated by 108 AAs can be categorized as being permeable to Si(OH)_4_.

## 4. Trophic Level Two: Si Interactions with Phytophagous Insects

Plants are sessile organisms that are constantly targeted by different groups of pests, including insects [75]. As a result of 350 million years of coevolution, plants have developed two main categories of defenses: constitutive defenses, which are expressed by non-stressed plants; and inducible defenses, which are activated upon herbivory [76]. Induced defenses are mediated by a set of phytohormones that serve as primary signals in the defense of plants against insects [77,78]. They include jasmonic acid (JA), salicylic acid (SA) and ethylene (ET) as key components [79]. While SA-mediated defense responses are often related to piercing and sucking insects and pathogens, JA/ET mediated defenses are mainly involved after chewing insect attacks and infestation by necrotroph pathogens [80]. These three phytohormones work individually, synergistically or antagonistically, according to the type of damage [81]. Si is involved in a very complex and critical role with different effects on multiple phytohormones working to alleviate biotic stresses [82].

Induced by Si accumulation, higher levels of JA, SA and ET have been observed in plants experiencing different host-pathogen interactions [26,83,84]. Accumulated Si has also the potential to modulate signal transduction of abscisic acid (ABA) involved in stress tolerance for plants [85]. In response to biotic stresses, cross talks between JA/ET, SA and ABA must occur and regulate induced defenses [86]. However, details concerning the relationship between those phytohormones and Si remains unclear.

Si was recently shown to have a strong interaction with jasmonates (i.e., JA and its derivatives), and associated plant defenses against herbivorous insects [87]. In maize seedlings exposed to cold stress, Si treatments restored phytohormones concentrations (JA, SA and ET) at comparable levels to those of unstressed plants [88]. In rice, Si primes jasmonate-mediated antiherbivore defense. By silencing the expression of two key enzymes (allene oxide synthase OsAOS associated with JA biosynthesis; and Coronatine Insensitive 1 OsCOI1 associated with JA perception) in transgenic rice plants via RNAi, Ye et al. [87] observed increased resistance against caterpillar (*Cnaphalocris medinalis* Guenée) in wild rice. Increasing Si concentrations in plants enhance JA accumulation and activities of two defense-related enzymes, namely peroxidase (POD) and polyphenol oxidase (PPO), as well as trypsin protease inhibitor (TrypPI). Si is also involved in the regulation of JA biosynthesis during a wounding stress [89]. Those results suggest an interplay between JA and Si in which Si enhances or primes JA-inducible responses to herbivory including the enhanced induction of defense-related enzymes and proteins, and enhanced induction of transcripts encoding proteins involved in JA signaling [87]. A very recent study confirmed this interplay between Si and JA in plants accumulating high levels of Si [90]: Using wild type (WT) and silicon-deficient mutant OsLsi1 rice plants, they showed that the expression levels of JA dependent genes (OsLOX, OsAOS2, OsCOI1a, OsCOI1b and OsBBPI) were much higher in Si-treated WT plants after infestation by *C. medinalis*, including transcripts encoding proteins involved in JA signaling, and defenses-related enzymes (catalase (CAT), superoxide dismutase (SOD) polyphenol oxidase (PPO) and peroxidase (POD). Interestingly, significant decreases in activity of defense-related enzymes and transcript levels were observed in Si transporter deficient mutant. Those results were correlated with a loss of *C. medinalis* resistance by mutant plants. Taken together, these findings suggest a strong interaction between Si and JA, with Si amplifying the response of plants mediated by induced defense. Recently, a conceptual model based on Si-JA interactions was proposed to evaluate the role of Si on antiherbivore phytohormonal signaling. In this perspective article, they support the hypothesis that silicon acts as a physical barrier interfering with plant’s infestation by herbivores [91]. 

In contrast, the effect of Si on ET is not clearly demonstrated. ET is another important hormone involved in plant responses to microbial pathogens and herbivorous insects, and in the interaction of plants with beneficial microbes and insects [92]. Moreover, ET signaling pathway works synergistically or antagonistically with JA [93], and has the potential to alter the HIPV blend of a plant. Kim et al. [94] did not observe modification of ET production after the application of exogenous Si on rice plants. However, ET production was significantly reduced in Si-rich plants submitted to wounding stresses compared to the non-amended plants. These results suggest possible interactions between ET and Si that deserve to be studied more deeply.

Vivancos et al. [26] studied the protective effect of Si on *Arabidopsis* mutants against powdery mildews. Their results clearly showed increasing SA concentrations in Si-enriched plants and an increase in expression of genes encoding enzymes involved in the SA pathway. Again, the mechanism responsible for this interaction deserves more efforts.

Inducible defenses include the synthesis and release of volatile organic molecules that are specifically produced under herbivore attack and called herbivore-induced plant volatiles (HIPVs). HIPVs can warn a neighboring undamaged plants of the upcoming danger, they can be used by the same plant to communicate among its different parts or even act as feeding or oviposition deterrents to pests [95,96,97]. For a pest natural enemy, HIPVs are also good indicators of the presence of preys [98]. Si may trigger different plant species to emit, amplify and/or alter HIPVs. Si has the potential to modulate HIPVs through priming JA defenses [87] and could also modulate other hormones involved in the production of HIPVs such as ET or SA. Si-treated cucumber plants infested by a chewing herbivore (*Diabrotica balteata* LeConte) produced more indole than the control infested plants, suggesting that the defensive pathways of Si-enriched plants are more likely to be primed than control ones, since indole is well known for plant defense priming [99]. Si accumulation in plants was shown to change grapevine (*Vitis vinifera* L.) volatile profile, with Si-treated plants infested by grapevine moth (*Phalaenoides glycinae* Lewin) producing higher amounts of n-heptadecane than control plants. In contrast, Cis-thio rose oxide production was significantly lower in silicon-treated grapevines [100]. Plants supplied with Si and infested by the rice leaf folder (*C. medinalis* Guenée) produced smaller amounts of hexanal-2-ethyl, α-bergamotene, β-sesquiphellandrene and cedrol than infested non-treated rice plants [23]. The way by which Si in plants alters HIPVs production is also not fully understood.

## 5. Trophic Level Three: Effect of Silicon on Natural Enemies of Pests

Induced chemical defenses include the emissions of HIPVs, whose function includes the guidance of natural enemies (predators or parasitoids) to their prey or hosts [101]. Kvedaras et al. [22] demonstrated that Si-enriched cucumber plants (*Cucumis sativus* L.) infested by cotton bollworm (*Helicoverpa armigera* Hübner) are more attractive to red and blue beetles (*Dicranolaius bellulus* Boisduval) than control plants. Moreover, using *H. armigera* eggs affixed to potted cucumber plants in a small-scale field trial, just before they were placed in a field plot of lucerne, was shown that increased biological control by “wild” predators was significantly higher for silicon-treated plants than for control plants. Connick et al. [100] observed a positive relationship between Si concentration in plant tissue and the level of attraction of the predatory beetle (*D. bellulus* Boisduval) to plants infested by light brown apple moths (*Epiphyas postvittana* Walker). When rice plants (*O. sativa*) are infested by the rice leaf folder (*C. medinalis* Guenée), Si-treated plants were more attractive to two parasitoid wasps (*Trathala flavor-orbitalis* Cameron and *Microplitis mediator* Haliday) [23].

## 6. Concluding Remarks and Perspectives

In light of the presented work, many tracks seem interesting to explore the effect of silicon on plant defenses. Given that plant’s Si accumulation is influenced by climatic and environmental parameters, further research should combine those variables to better evaluate the potential of Si application as component of future integrated pest management. In addition, the soil-plant system should receive additional attention, especially the diversity of agricultural soils, under different climatic conditions. 

Plants do not all have the same ability to assimilate bioavailable silicon. One should compare the effect of Si fertilization on monocots and dicots and/or on Si-accumulators and non-accumulators species. Silicon is involved in the plant’s response against attack of a phytophagous insect by modifying expression of genes encoding for defensive enzymes. Further studies on the interactions of Si with the transcriptome analysis of different plant species varying in their Si uptake ability under attack of different forms of insect herbivory should provide valuable insight into how Si alters plant gene expression implicated in HIPVs production. Given Si alters volatile blends in different ways, it is central to increase our understanding of the Si-mediated mechanisms that affect the composition of the volatile blend. Si influences HIPVs’ release by priming phytohormones (JA/ET, SA) signaling pathways, but their network interactions are very complex and the influence of Si in regulating plant volatile blends is not understood. Given the fact that there is a strong interaction between Si and JA, studying JA metabolites such as (+)-12-oxo-phytodienoic acid (OPDA) or methyl jasmonate (meJA) could also bring additional information on this topic.

As part of their defense mechanisms, plants emit HIPVs that serves as cues for natural enemies to locate insect pests. The role of Si on the emission of HIPVs could improve the ability of pest’s natural enemies to locate their prey or hosts. Given the few studies conducted so far and the gigantic number of possibilities for plant specific interactions with pests and natural enemies, silicon impact on tritrophic relationship should be tested for different plant models. When evaluating the volatile organic compounds (VOCs) emission of one plant, one should also compare the impact of various types of insect herbivory. Plants can communicate on vertical channels between below- and aboveground plant-feeding insects. Aboveground herbivores impact the development and growth of plant’s feeders but also the attractiveness of the parasitoid of leaf feeders. The underlying mechanism mediating this effect is based on changes in the volatile blend of the plant [102]. It would be useful to study the combined effect of a root pest and silicon on the attractiveness of the plant to parasitoids. It remains to clarify how silicon changes a plant volatilome, and what organisms are impacted, whether they belong to the second, third or fourth trophic level of the food chain.

Among the emerging strategies of pest control lies the use of Si supplement to soil. Indeed, there is now a large number of studies suggesting the high effectiveness of Si to promote physical and biochemical plant direct defense against insect herbivores [103,104]. This review attempted to gather current knowledge on an important part of induced indirect plant defenses: the promotion of plant volatile emissions. Few studies evaluate the impact of silicon on HIPVs’ emission by plants, it would be interesting to increase their numbers and look into the possibility of including silicon as a new weapon in crop protection and pest management [105].
